# Evaluating the Use of ChatGPT 3.5 and Bard as Self-Assessment Tools for Short Answer Questions in Undergraduate Ophthalmology

**DOI:** 10.7759/cureus.86288

**Published:** 2025-06-18

**Authors:** Abhijeet M Khake, Suvarna Gokhale, Pradeep Dindore, Sonali Khake, Manjiri Desai

**Affiliations:** 1 Department of Ophthalmology, Pacific Medical College and Hospital, Udaipur, IND; 2 Department of Ophthalmology, Smt. Kashibai Navale Medical College and General Hospital, Pune, IND; 3 Department of Ophthalmology, Prakash Institute of Medical Sciences and Research, Uran Islampur, IND; 4 Department of Anatomy, SSPM Medical College and Lifetime Hospital, Sindhudurg, IND; 5 Department of Community Medicine, D. Y. Patil Medical College, Kolhapur, IND

**Keywords:** artificial intelligence, chatgpt 3.5, deep learning, large language models, ophthalmology, self-assessment, short answer questions, undergraduate medical education

## Abstract

Objective: This study aimed to evaluate the efficacy of ChatGPT 3.5 and Google Bard as tools for self-assessment of short answer questions (SAQs) in ophthalmology for undergraduate medical students.

Methodology: A total of 261 SAQs were randomly selected from previous university examination papers and publicly available ophthalmology question banks. The questions were classified according to the competency-based medical education (CBME) curriculum of the National Medical Commission (NMC) of India into three categories: short note task-oriented questions (SNTO, n = 169), short note reasoning questions (SNRQ, n = 15), and applied aspect SAQs (SN Applied, n = 77). Image-based questions were excluded. Three ophthalmologists collaboratively developed model answers for each question. The same questions were then submitted to ChatGPT 3.5 and Google Bard. The AI-generated responses were independently evaluated by three ophthalmologists using a 3-point scale based on correct diagnosis, accuracy of content, and relevance. The scores were compiled, and the data were analyzed to compare the overall and category-wise performance of the two AI tools.

Results: Out of a total possible score of 783 (261 questions × 3 points), ChatGPT 3.5 scored 696 (88.8%), while Bard scored 685 (87.5%). Although the overall performance difference was insignificant, ChatGPT 3.5 performed significantly better in the SNTO category. However, both AI tools produced poor-quality or inadequate answers for a subset of questions: 50 (19%) by ChatGPT 3.5 and 44 (16.8%) by Bard. Some responses lacked essential information, even for high-yield topics.

Conclusion: ChatGPT 3.5 and Bard can generate accurate and relevant responses to ophthalmology SAQs in most cases. ChatGPT 3.5 demonstrated slightly better performance, particularly for task-oriented questions, suggesting it may be a more effective tool for undergraduate students’ self-assessment. However, due to a notable error rate (~20%), AI-generated responses should not be used in isolation and must be cross-referenced with standard textbooks. These tools best suit rapid information retrieval during the early study phases.

## Introduction

The integration of artificial intelligence (AI) and deep learning (DL) into medical practice has gained momentum over the past decade, with ophthalmology being one of the earliest specialties to adopt these technologies [1]. DL has shown significant efficacy in identifying retinal disorders from fundus photographs and OCT images, particularly in diabetic retinopathy and glaucoma [2,3]. Based on these findings, several AI-based diagnostic tools have been developed.

Natural language processing (NLP), a branch of AI focused on understanding and interacting with human language, has also gained considerable attention in ophthalmology. This has led to the development of large language models (LLMs) such as OpenAI’s ChatGPT and Google’s Bard [4]. The use of these LLMs is now being investigated in medical education. Recent studies have reported that ChatGPT has successfully passed medical licensing examinations, including the United States Medical Licensing Examination (USMLE) [5].

Inspired by these advancements, researchers have begun exploring the application of LLMs in assessing undergraduate medical students from various perspectives [6-10]. These models have already been evaluated as tools for student assessment in subjects such as internal medicine, pulmonology, physiology, biochemistry, microbiology, and pathology [7-13]. However, their use in ophthalmology education remains underexplored.

Undergraduate medical students typically gain theoretical knowledge through textbooks, didactic lectures (both in-person and online), small group discussions, and bedside clinical teaching. Their knowledge and skills are evaluated through periodic assessments [6,8]. Theoretical evaluations often consist of long- and short-answer questions - including structured clinical or clinical reasoning components - and multiple-choice questions. Students rely on textbooks, past exam papers, and online learning platforms for self-assessment [6].

The emergence of LLMs such as ChatGPT and Google Bard has made access to relevant information easier and more immediate. While conventional search engines such as Google provide web links for information retrieval, LLMs can generate direct and concise answers, saving time and effort. Furthermore, their interactive nature suggests potential use as evaluation tools. However, since these models are not specifically designed for medical education in ophthalmology, evaluating their reliability and accuracy is essential before recommending them for undergraduate self-assessment [14-17].

Short-answer questions (SAQs) are a valuable assessment method because they can cover a wide range of content within a limited timeframe. According to competency-based medical education (CBME) guidelines, SAQs in clinical subjects may be task-oriented, reasoning-based, or focused on applied aspects of preclinical topics as per Annexure 2 of Module 3 (Assessment Module) for undergraduate medical education 2019 by NMC [18]. These questions allow efficient and targeted evaluation of knowledge and comprehension across a broad subject area. This study aims to investigate the efficacy of ChatGPT 3.5 and Google Bard as instruments for the self-evaluation of SAQs in ophthalmology for undergraduate students.

## Materials and methods

Question selection and categorization

A total of 261 short answer questions were randomly selected from previous university examination papers and publicly available ophthalmology question banks. A computer-generated random number table was used to ensure an unbiased and objective selection of questions from a larger compiled pool. These questions were classified according to the competencies outlined in the CBME curriculum prescribed by the NMC, India. Image-based and multiple-choice questions were excluded from the study.

The SAQs were categorized into three groups: task-oriented SAQs (SNTO), short answer reasoning questions (SNRQ), and short answer applied aspect questions (SN Applied). Task-oriented SAQs are clinical scenario-based and are designed to assess knowledge and comprehension related to specific aspects of a clinical problem. These questions require students to mentally or physically perform tasks to address the clinical scenario. Examples include questions such as “How will you manage this case?”, “What is your immediate plan of treatment?”, “What investigations will you order?”, “What clinical signs will you elicit?”, or “How will you identify a complication in this case?”.

SNRQ assess clinical reasoning, integration, and analytical ability. These may be either straightforward or scenario-based. Examples include the following: “What differential diagnoses would you consider for this case?”, “Why would you select a specific treatment modality?”, “What additional history is needed to reach a diagnosis?”, or “Why is a particular investigation appropriate?”.

Short answer applied aspect questions evaluate the foundational knowledge of preclinical subjects in clinical contexts. These may also be either straightforward or scenario-based. Examples include questions about the etiopathogenesis of a condition, the mechanism of action of a drug, the anatomical or physiological basis of a symptom, or the microbiological or pathological findings of a given disease. Of the 261 questions, 169 were SNTO, 15 were SNRQ, and 77 were SN Applied.

Development of model answers

Three experienced ophthalmologists collaboratively prepared model answers for all 261 questions. Standard reference materials, including the 24th edition of Parsons’ Diseases of the Eye and EyeWiki, an online educational platform developed by the American Academy of Ophthalmology, were used to guide the formulation of these responses [19]. The finalized model answers served as the standard against which AI-generated answers were evaluated.

Evaluation of AI-generated responses

Each of the 261 questions was submitted to ChatGPT 3.5 and Google Bard. The responses generated by each tool were independently evaluated by three ophthalmologists based on content accuracy, conceptual clarity, and relevance to the question. A 3-point scoring system was used. A score of 1 was assigned if the response did not align with the standard, contained incorrect concepts, or deviated significantly from accepted medical knowledge. A score of 2 was given for partially correct answers, where the core concept was valid but essential details were missing or less than 80% of the key content was covered. A score of 3 indicated a high-quality response that fully aligned with the standard, demonstrated accurate conceptual understanding, and covered more than 80% of the essential content.

For each question, the final score for an AI-generated response was determined by consensus among the evaluators, which was defined as agreement between at least two of the three ophthalmologists. Individual scores were not reported separately; only the consensus score was considered for analysis. Significantly, these scores were not influenced by the model answers, which were developed independently and before AI evaluation.

Classification of response quality

In addition to numerical scoring, AI-generated responses were qualitatively classified into “good” or “poor” responses to assess their suitability for undergraduate medical education. Responses were classified as “good” if conceptually accurate and included more than 90% of the key information. Responses were classified as “poor” if conceptually incorrect or contained less than 50% of essential content. Responses falling between 50% and 90% content coverage were not categorized into either group but remained part of the overall scoring analysis. This dichotomous classification was used to better understand the educational quality of responses generated by each AI tool. All data, including scores and classifications, were systematically recorded in an Excel spreadsheet (Microsoft® Corp., Redmond, WA) and analyzed based on the type of SAQs - SNTO, SNRQ, or SN Applied.

Statistical analysis

Statistical analysis was done using Statistical Product and Service Solutions (SPSS, version 25; IBM SPSS Statistics for Windows, Armonk, NY). Statistical analysis was carried out for topic-wise comparisons and for comparing the proportion of good-quality answers between AI tools, using the Z test for proportions. A p-value of less than 0.05 was considered statistically significant.

## Results

A total of 261 SAQs were analyzed, comprising 169 (64.8%) SNTO, 15 (5.7%) SNRQ, and 77 (29.5%) SN Applied.

Table 1 shows that, for task-oriented SAQs, the accuracy and adequacy of information score provided by ChatGPT were 456 out of 507 (89.9%), while Bard achieved 449 out of 507 (88.6%). Both ChatGPT and Bard were able to generate responses with commendable precision. ChatGPT delivered satisfactory responses in 40 (23.7%) cases, whereas Bard did so in 11 (6.5%) cases, indicating that ChatGPT was superior in generating more precise and detailed answers. However, ChatGPT produced significantly incorrect responses - unsuitable for undergraduate medical education - in 31 (18.3%) cases, while Bard did so in 29 (17.1%) cases. This indicates that, although ChatGPT performs better overall, it can produce significant errors approximately once in every five responses.

**Table 1:** Short answer task-oriented questions (SNTO)

Subject	Number of questions	Chat GPT score (Total score/Max score)	Bard score total score/max score	No. of good answers		No. of bad answers	
				Chat GPT	Bard	Chat GPT	Bard
Conjunctiva	17 (10.05%)	45/51	49/51	5	3	3	1
Cornea	32 (18.9%)	88/96	82/96	9	4	4	3
Iris & anterior chamber	29 (17.1%)	80/87	78/87	7	1	5	5
Lens	10 (5.9%)	24/30	21/30	1	0	4	5
Lids, adnexa and orbit	20 (11.83%)	53/60	56/60	4	1	3	3
Retina and optic nerve	30 (17.7%)	84/90	80/90	10	2	4	5
Sclera	5 (2.9%)	15/15	14/15	0	0	0	0
Miscellaneous	19 (11.24%)	54/57	53/57	4	0	3	3
Visual acuity assessment	7 (4.14%)	13/21	16/21	0	0	5	4
Total	169 (100%)	456/507	449/507	40 (23.66%)	11 (6.50%	31 (18.34%)	29 (17.15%)

Table 2 indicates that, for reasoning-based questions, both ChatGPT and Bard had an equal accuracy rate score 39 out of 45 (86.7%). Thus, the two tools demonstrated comparable performance in this category. However, ChatGPT generated significantly incorrect responses in four (26.7%) cases, whereas Bard did so in two (13.3%) cases. This suggests that ChatGPT may provide more comprehensive content, but also carries a higher risk of critical inaccuracies - about one in every four responses.

**Table 2:** Distribution of short answer reasoning questions (SNRQ)

Topic	No. of Questions	Chat GPT score (Total score/Max score)	Bard score (Total score/Max score)	No of good answers		No of bad answers	
				Chat GPT	Bard	Chat GPT	Bard
Conjunctiva	0 (0%)	-	-	-	-	-	-
Cornea	3 (20%)	9/9	8/9	0	0	0	0
Iris and anterior chamber	1 (6.6%)	3/3	3/3	0	0	0	0
Lens	1 (6.6%)	1/3	2/3	0	0	1	0
Retina and optic nerve	8 (53.3%)	21/24	21/24	0	0	2	1
Miscellaneous	1 (6.6%)	3/3	3/3	0	0	0	0
Visual acuity assessment	1 (6.6%)	2/3	2/3	0	0	1	1
Total	15 (100%)	39/45	39/45	0 (0%)	0 (0%)	4 (26.66%)	2 (13.33%)

Table 3 shows that ChatGPT provided an accurate and adequate information score for applied aspect questions of 201 out of 231 (87.0%) responses, while Bard achieved this in 197 out of 231 (85.7%) responses. Both tools demonstrated good overall performance. ChatGPT delivered satisfactory responses in 12 (15.6%) cases, compared to seven (9.1%) cases by Bard, again highlighting its relatively better precision. However, ChatGPT also gave significantly erroneous answers in 15 (19.5%) cases, compared to 13 (16.9%) by Bard. This underscores that ChatGPT may still produce significant errors in about one out of five instances despite higher precision.

**Table 3:** Distribution of short answer question applied aspect (SN Applied)

Topic	No. of questions	Chat GPT Score (Total score/Max score)	Bard Score (Total score/Max score)	No. of good answers		No. of bad answers	
				Chat GPT	Bard	Chat GPT	Bard
Conjunctiva	6 (7.7%)	14/18	17/18	0	2	1	0
Cornea	12 (15.5%)	34/36	33/36	2	3	1	1
Iris and anterior chamber	10 (12.9%)	27/30	25/30	2	0	2	2
Lens	6 (7.7%)	16/18	16/18	1	1	2	2
Lids, adnexa and orbit	9 (11.6%)	25/27	24/27	1	0	1	1
Retina and optic nerve	18 (23.3%)	45/54	42/54	5	1	4	3
Miscellaneous	14 (18.1%)	35/42	35/42	1	0	3	3
Visual acuity assessment	2 (2.5%)	5/6.	5/6	0	0	1	1
Total	77 (100%)	201/231	197/231	12 (15.6%)	7 (9.09%)	15 (19.48%)	13 16.88%)

Table 4 presents a topic-wise comparison between ChatGPT 3.5 and Bard. A statistically significant difference in total scores was observed only in conjunctiva (p = 0.041), where Bard outperformed ChatGPT 3.5. No statistically significant differences were found when overall scores were compared for all other topics.

**Table 4:** Topic-wise score comparison p < 0.05 is considered statistically significant; SNRQ: short answer reasoning questions; SNTO: short answer task-oriented questions

Topic	Question type	No. of Questions	Chat GPT Score	Bard Score	p-value
			Total score/Max score	Total score/Max score	
Conjunctiva	SN applied	6	14/18	17/18	0.14
	SN RQ	-	-	-	-
	SNTO	17	45/51	49/51	0.14
	Total	23	59/69	66/69	0.041*
Cornea	SN applied	12	34/36	33/36	0.64
	SN RQ	3	9/9.	8/9.	0.3
	SNTO	32	88/96	82/96	0.17
	Total	47	131/141	123/141	0.11
Iris and the anterior chamber	SN applied	10	27/30	25/30	0.44
	SN RQ	1	3/3.	3/3.	1
	SNTO	29	80/87	78/87	0.6
	Total	40	110/140	106/140	0.56
Lens	SN applied	6	16/18	16/18	1
	SN RQ	1	1/3	2/3	0.41
	SNTO	10	24/30	21/30	0.37
	Total	17	41/51	39/51	0.63
Lids, adnexa and orbit	SN applied	9	25/27	24/27	0.63
	SN RQ	-	-	-	-
	SNTO	20	53/60	56/60	0.34
	Total	29	78/87	80/87	0.6
Miscellaneous	SN applied	14	35/42	35/42	1
	SN RQ	1	3/3	3/3	1
	SNTO	19	54/57	53/57	0.69
	Total	34	92/102	91/102	0.81
Retina and optic nerve	SN applied	18	45/54	42/54	0.46
	SN RQ	8	21/24	21/24	1
	SNTO	30	84/90	80/90	0.29
	Total	56	150/168	143/168	0.25
Visual acuity assessment	SN applied	2	5/6	5/6	1
	SN RQ	1	2/3	2/3	1
	SNTO	7	13/21	16/21	0.31
	Total	10	20/30	23/30	0.38
Total	SN applied	77	201/231	197/231	0.58
	SN RQ	15	39/45	39/45	1
	SNTO	169	456/507	449/507	0.47
	Total	261	696/783	685/783	0.38

Table 5 summarizes the comparison of good-quality responses provided by ChatGPT 3.5 and Bard. A statistically significant difference was found in the total scores for the iris and anterior chamber and retina (p < 0.05), with ChatGPT 3.5 outperforming Bard. Overall, ChatGPT 3.5 generated a significantly higher number of correct responses than Bard.

**Table 5:** Good answer comparison p < 0.05 is considered statistically significant; SNRQ: short answer reasoning questions; SNTO: short answer task-oriented questions

Topic	Question type	No. of questions	Chat GPT	Bard	p-value
Conjunctiva	SN applied	6	0	2	0.12
	SN RQ	-	-	-	
	SNTO	17	5	3	0.41
	Total	23	5	5	1
Cornea	SN applied	12	2	3	0.61
	SN RQ	3	0	0	-
	SNTO	32	9	4	0.12
	Total	47	11	7	0.29
Iris and anterior chamber	SN applied	10	2	0	0.13
	SN RQ	1	0	0	
	SNTO	29	7	1	0.022
	Total	40	9	1	0.0069**
Lens	SN applied	6	1	1	1
	SN RQ	1	0	0	-
	SNTO	10	1	0	0.3
	Total	17	2	1	0.54
Lids, adnexa and orbit	SN applied	9	1	0	0.3
	SN RQ	-	-	-	-
	SNTO	20	4	1	0.085
	Total	29	5	1	0.15
Miscellaneous	SN applied	14	1	0	0.3
	SN RQ	1	0	0	-
	SNTO	19	4	0	0.034
	Total	34	5	0	0.020
Retina and optic nerve	SN applied	18	5	1	0.073
	SN RQ	8	0	0	
	SNTO	30	10	2	0.0098
	Total	56	15	3	0.002**
Visual acuity assessment	SN applied	2	0	0	-
	SN RQ	1	0	0	-
	SNTO	7	0	0	-
	Total	10	0	0	-
Total	SN applied	77	12	7	0.21
	SN RQ	15	0	0	
	SNTO	169	40	11	p<0.0001
	Total	261	52	18	p<0.0001

## Discussion

Our study aimed to determine whether LLMs, such as OpenAI’s ChatGPT 3.5 and Google’s Bard, can be self-assessment tools for undergraduate-level SAQs in ophthalmology. A total of 261 SAQs were selected. Of these, 169 were SNTO, 15 were SNRQ, and 77 were SN Applied. These SAQs were posed to ChatGPT 3.5 and Bard. The overall scores for accuracy and adequacy of information were 696 out of 783 (88.8%) for ChatGPT 3.5 and 685 out of 783 (87.5%) for Bard. The difference between the two tools was not statistically significant. However, a statistically significant difference was observed in the number of correct responses in the SNTO category, with ChatGPT 3.5 outperforming Bard.

Additionally, ChatGPT 3.5 and Bard provided incorrect answers for 50 out of 261 questions (19.2%) and 44 out of 261 questions (16.9%), respectively. Furthermore, several responses on high-yield topics lacked essential details in both tools. Our analysis indicated that, while both ChatGPT 3.5 and Bard frequently delivered accurate and adequate responses, ChatGPT 3.5 performed better overall and is more suitable as a self-assessment tool for SAQs. Nevertheless, ChatGPT was grossly inaccurate in nearly one out of five responses, so it should not be used in isolation. Responses must be cross-verified with standard textbooks.

In their current form, ChatGPT 3.5 and Bard are best utilized for rapid information retrieval during the early phases of undergraduate medical education. The undergraduate curriculum is dense and primarily focused on equipping students with essential knowledge and the ability to think critically and compassionately. The prevailing view is that comprehensive AI integration is better suited for postgraduate or fellowship-level training when students have a more developed understanding of clinical decision-making and workflows [20].

LLMs are simple to use, require no additional training, and can be integrated at the undergraduate level. Agarwal et al. [9] found that LLMs, such as ChatGPT, Bard, and Bing, could evaluate questions of varying difficulty in physiology; however, they remain underdeveloped and have inherent limitations. Additional studies suggest that ChatGPT is useful for tasks requiring higher-order cognitive functions - such as interpretation, analysis, evaluation, and evidence-based opinion formation - in subjects such as pathology, biochemistry, and microbiology [10-12]. Surapaneni et al. [13] evaluated ChatGPT’s utility as a self-directed learning tool in medical biochemistry and scored only 58% on an examination paper, indicating room for improvement.

Significantly, our study was limited to earlier-generation models - GPT-3.5 and Bard - which do not utilize tool-use features or deep web searches. In contrast, newer models such as GPT-4, Gemini (Bard Advanced), and Med-PaLM 2 are expected to significantly improve contextual reasoning, accuracy, and domain-specific clinical relevance [21-23]. These newer models also support real-time information retrieval and deeper semantic search capabilities, potentially reducing the error rate observed in our study. However, these enhancements may come at the cost of speed, complexity, or accessibility, essential trade-offs in the undergraduate learning environment.

We also acknowledge that the dichotomous classification of AI responses as “good” (>90% content accuracy) and “bad” (<50% content accuracy) may not fully capture the educational nuance. Although it helped in statistical clarity, such thresholds are arbitrary and not validated by prior literature. Pedagogically, flawed or partial answers might offer value by encouraging student reflection and error correction. Studies have shown that even incorrect AI-generated content can be helpful as a scaffold for learning. For instance, Pardos et al. [24] demonstrated that students using ChatGPT-generated algebra hints still exhibited measurable learning gains, even when hints were less practical than those generated by human tutors. Dahlkemper et al. [25] reported that physics students could assess and sometimes accept flawed ChatGPT responses, underscoring the importance of student agency and reflective evaluation. A recent meta-analysis on chatbot use in STEM education also found that guided use of AI, even when imperfect, was associated with improved learning outcomes [26]. Moreover, Ali et al. [27] emphasized that AI applications foster creativity and personalized learning even when outputs are incomplete or incorrect, especially when integrated within thoughtful instructional design frameworks. These findings suggest that educators and students might benefit more from a nuanced grading system - such as excellent, acceptable, incomplete, or misleading - than a strict binary.

Limitations

There is limited evidence regarding the use of LLMs in clinical settings. This study aimed to bridge that gap in the field of ophthalmology. ChatGPT 3.5 was a comparatively better tool for SAQ assessment than Bard, but still has a one-in-five chance of producing significantly inaccurate responses. Its use should remain supplementary to standard textbooks during self-assessment. We recommend that its application be restricted to early-stage learning for rapid and approximate self-evaluation by undergraduate medical students in ophthalmology. We anticipate that these tools will be further refined to yield more accurate responses - ideally above 90% - or that specialized LLMs will be developed to provide updated and highly reliable content for the medical profession.

This study used ChatGPT 3.5 and Bard (original version), as these were the only LLMs available during the study period (late 2023 to early 2024). Newer models, such as ChatGPT-4 and Gemini, were launched only after data collection had concluded. The absence of these advanced tools is a limitation when viewed from the present timeline. Given their improved clinical accuracy, contextual reasoning, and use of dynamic search capabilities, their performance may differ significantly and warrants future evaluation.

Additionally, our classification of AI-generated responses into “good” or “bad” was not based on prior literature or externally validated by a panel of ophthalmologists. These categories were derived from comparison with model answers and served operational simplicity rather than being pedagogically comprehensive. This limits generalizability and may overlook nuanced learning potential embedded in partially correct responses.

## Conclusions

Our study explored the utility of LLMs, specifically ChatGPT 3.5 and Bard, as self-assessment tools for undergraduate ophthalmology SAQs. Both models demonstrated the potential to deliver contextually relevant and pedagogically useful responses, with ChatGPT 3.5 showing slightly superior performance in specific question categories. However, occasional inaccuracies and omissions highlight the need for careful cross-verification with standard educational resources. These tools are best used as supplementary aids during early medical education rather than as standalone learning platforms. As AI technologies evolve, more advanced models may offer enhanced reliability, and their integration into medical education warrants further exploration.
